# Increased platelet activation and platelet-inflammasome engagement during chikungunya infection

**DOI:** 10.3389/fimmu.2022.958820

**Published:** 2022-09-15

**Authors:** Isaclaudia Gomes de Azevedo-Quintanilha, Mariana Macedo Campos, Ana Paula Teixeira Monteiro, Alessandra Dantas do Nascimento, Andrea Surrage Calheiros, Douglas Mathias Oliveira, Suelen Silva Gomes Dias, Vinicius Cardoso Soares, Julia da Cunha Santos, Isabel Tavares, Thiago Moreno Lopes Souza, Eugenio D. Hottz, Fernando A. Bozza, Patricia T. Bozza

**Affiliations:** ^1^ Laboratório de Imunofarmacologia, Instituto Oswaldo Cruz, Fundação Oswaldo Cruz, Rio de Janeiro, Brazil; ^2^ Instituto Nacional de Infectologia Evandro Chagas, Fundação Oswaldo Cruz, Rio de Janeiro, Brazil; ^3^ Instituto D’Or de Pesquisa e Ensino, Rio de Janeiro, Brazil; ^4^ Centro de Desenvolvimento Tecnológico em Saúde (CDTS) and National Institute for Science and Technology on Innovation on Diseases of Neglected Populations (INCT/IDNP), FIOCRUZ, Rio de Janeiro, Brazil; ^5^ Laboratório de Imunotrombose, Departamento de Bioquimica, Universidade Federal de Juiz de Fora, Juiz de Fora, Brazil

**Keywords:** platelets, activation, chikungunya, inflammatory mediator, inflammasome

## Abstract

Chikungunya fever is a viral disease transmitted by mosquitoes of the genus Aedes. The infection is usually symptomatic and most common symptoms are fever accompanied by joint pain and swelling. In most cases symptoms subside within a week. However, severe prolonged and disabling joint pain, that may persist for several months, even years, are reported. Although the pathogenesis of Chikungunya infection is not fully understood, the evolution to severe disease seems to be associated with the activation of immune mechanisms and the action of inflammatory mediators. Platelets are recognized as inflammatory cells with fundamental activities in the immune response, maintenance of vascular stability and pathogenicity of several inflammatory and infectious diseases. Although the involvement of platelets in the pathogenesis of viral diseases has gained attention in recent years, their activation in Chikungunya has not been explored. The aim of this study was to analyze platelet activation and the possible role of platelets in the amplification of the inflammatory response during Chikungunya infection. We prospectively included 132 patients attended at the Quinta D’Or hospital and 25 healthy volunteers during the 2016 epidemic in Rio de Janeiro, Brazil. We observed increased expression of CD62P on the surface of platelets, as well as increased plasma levels of CD62P and platelet-derived inflammatory mediators indicating that the Chikungunya infection leads to platelet activation. In addition, platelets from chikungunya patients exhibit increased expression of NLRP3, caspase 4, and cleaved IL-1β, suggestive of platelet-inflammasome engagement during chikungunya infection. *In vitro* experiments confirmed that the Chikungunya virus directly activates platelets. Moreover, we observed that platelet activation and soluble p-selectin at the onset of symptoms were associated with development of chronic forms of the disease. Collectively, our data suggest platelet involvement in the immune processes and inflammatory amplification triggered by the infection.

## Introduction

Chikungunya virus (CHIKV) has a genome composed of single stranded RNA, being a member of the genus Alphavirus, and is transmitted by the bite of mosquitos of Aedes genus (1). Chikungunya fever outbreaks have already occurred in parts of Africa, Europe, Southeast Asia, the islands of the Indian and Pacific oceans. In 2013 it was first reported in America and has now been identified in the Caribbean and North, Central and South America (2, 3). Nowadays, Chikungunya is a public health problem in Brazil, with 90,000 cases in 2021 corresponding to an increase of 32.6% related to the previous year (4). CHIKV infection is usually self-limiting, non-fatal, with fever resolving within a few days. Chikungunya fever’s most notable clinical feature is the persistent musculoskeletal symptoms for weeks, months, and even years. Patients may evolve with chronic polyarthritis, which resemble autoimmune inflammatory arthritis, with the pathogenesis not yet fully understood (5).

It has been demonstrated that CHIKV replication occurs in fibroblasts both *in vitro* and in target tissues (muscle, connective tissue and skin) of mice and humans (6, 7). Viral replication triggers the activation of innate immune responses, whose main characteristic is the production of type I interferons (IFNs) (8). Infection of primary human skin fibroblasts with CHIKV triggers, in addition to IFN-γ, increased expression of interleukin-1β (IL-1β), caspase-1 maturation, and expression of AIM2 inflammasome sensor. Caspase-1 silencing increased viral replication, suggesting that CHIKV-infected skin fibroblasts may contribute to a proinflammatory and antiviral microenvironment (9). Additionally, patients with acute chikungunya fever demonstrated increased levels of cytokines such as IL-1β, IL-6, tumor necrosis factor (TNF) and CCL-5/RANTES (10–13). CHIKV arthralgia is mostly characterized by severe joint pain associated with inflammation, tissue destruction and release of proinflammatory cytokines such as IL-1β, IL-6, TNF and 10 kDa IFN-induced protein (IP-10). Thus, it is plausible that CHIKV infection induces a self-perpetuation of the proinflammatory reaction that causes arthralgia years after recovery from the initial febrile phase (10, 11, 13–16).

Thrombocytopenia is a common feature in arboviral infections, especially in dengue (17). In CHIKV infection, symptoms such as hemorrhage and thrombocytopenia are considered atypical (18), but the role played by platelets in the inflammatory amplification during CHIKV infection remains unknown. Platelets are well known for their hemostatic activities. In addition to its role in thrombosis and hemostasis, platelets participate in other pathophysiological processes including inflammation, atherogenesis, host defense and tumor growth and metastasis (19, 20). Platelets have an extensive repertoire of surface receptors that transmit signals to their interior, and trigger signal transduction pathways (21, 22). Platelets express receptors capable of recognizing viral pathogens, as demonstrated for human immunodeficiency virus (HIV) (23), influenza vírus (24), hepatitis C virus (HCV) (25) and Dengue virus (DENV) (26). Platelets also mediate inflammatory and immunological processes that amplify the thromboinflammatory response, reprogramming adjacent cells and their functions. Platelet activation and platelet-leukocyte interactions are reflected in the pathophysiology of diseases, especially in inflammatory events by regulating the release of cytokines, extrusion of neutrophil extracellular traps (NETs), and monocytes and lymphocytes functions (27–29). When platelets are activated in pathological situations, they may contribute to the breakdown of the endothelial barrier, leading to fluid leakage and edema formation (21)

Our group demonstrated that platelets from patients acutely infected with dengue have evidence of activation, apoptosis and mitochondrial dysfunction (30). In addition, DENV infection activates platelets with subsequent effects on the vascular permeability and inflammation mediated by nucleotide- binding oligomerization domain (NOD) like receptor proteins 3 (NLRP3) and caspase-1 dependent secretion of IL-1β in platelet extracellular vesicles (31). Even though the contributions of platelets to inflammatory amplification and disease pathogenesis have been identified in several viral diseases including dengue, HCV, influenza and COVID-19, platelet responses in patients with chikungunya infection was not previously addressed. We hypothesized that platelets are activated and contribute to inflammatory amplification during Chikungunya infection. Here, we provide evidence that infection with chikungunya virus leads to increased platelet activation, and release of inflammatory mediators that may contribute to disease pathogenesis.

## Methods

### Human subjects

We prospectively enrolled 132 RT-PCR confirmed cases of acute Chikungunya fever in the Hospital Quinta D’Or in Rio de Janeiro, Brazil. Clinical data and peripheral venous blood samples were obtained. Molecular diagnosis was performed for chikungunya, dengue and zika, both in patient samples and in healthy donors. The research protocol used was approved by the Institutional Review Board (IOC/FIOCRUZ 42999214.1.1001.5248) and informed consent was obtained before any study related procedure. To perform the study, patients with CHIKV of both sexes, ranging in age from 18 to 65 years, were included. Patients who were infected with dengue, patients infected with another species of febrile illness, and pregnant women and patients who used nonsteroidal anti-inflammatory drugs up to 6 hours prior to blood collection were excluded. We have also included 25 healthy volunteers who were RT-PCR negative for chikungunya, dengue and zika viruses, did not present with fever or other symptoms and did not use non-steroidal anti-inflammatory drugs prior to blood draw.

### Platelet isolation

Peripheral blood samples were drawn into acid-citrate-dextrose (ACD) and centrifuged at 200 g for 20 min to obtain platelet-rich plasma (PRP). Briefly, PRP was centrifuged at 500 g for 20 min in the presence of 100 nM Prostaglandin E_1_ (PGE_1_) (Cayman Chemicals). The platelet pellet was resuspended in 2.5 mL of phosphate-buffered saline containing 2 mM EDTA, 0.5% human serum albumin, and 100 nM PGE_1_ and incubated with anti-CD45 tetrameric antibody complexes (1:25) for 10 min and with dextran-coated magnetic beads (1:50) for additional 10 min before purification in a magnet (Human CD45 depletion kit; Stem Cell, Easy Sep Technology). Recovered platelets were resuspended in 25 mL of PSG (PIPES-saline-glucose: 5 mM C_8_H_18_N_2_O_6_S_2_, 145 mM NaCl, 4 mM KCl, 50 mM Na_2_HPO_4_, 1 mM MgCl_2_-6H_2_O, and 5.5 mM glucose) containing 100 nM of PGE_1_. The platelet suspension was centrifuged at 500 g for 20 min. The supernatant was discarded, and the pellet was resuspended in medium 199 (Lonza). The platelet preparations purity (> 99% CD41+) was confirmed by flow cytometry.

### Plasma obtention

Plasma was obtained from ACD-anticoagulated blood by centrifuging the platelet-poor plasma (PPP) obtained above or citrate-anticoagulated blood at 900 x *g* for 20 min and stored at -80°C until use.

### Flow cytometry analysis

Platelets (1–5 x 10^6^) were incubated with FITC-conjugated anti-CD41 (BD Phamingen, CA) (1:20) and PE–conjugated anti-CD62P (BD Phamingen, CA) (1:20) for 30 min at 37°C. Isotype-matched antibodies were used to control the nonspecific binding of antibodies. Platelets were distinguished by specific binding of anti-CD41 and characteristic forward and side scattering. A minimum of 10,000 gated events was acquired using a FACScalibur flow cytometer (BD Bioscience, CA).

### Western blotting

Freshly isolated platelets from CHIKV patients and healthy volunteers were lysed [0.15 M NaCl, 10mM Tris pH 8.0, 0.1 mM EDTA, 10% (v/v) Glycerol and 0.5% (v/v) Triton X-100] in the presence of a protease inhibitor cocktail (Roche, Indianapolis, IN). Platelet proteins (25 μg) were separated by SDS-containing 15% polyacrylamide gel (SDS-PAGE) and transferred into nitrocellulose membranes. Membranes were blocked in Tris-buffered saline (TBS) supplemented with 0.1% Tween 20 (TBS-T) plus 5% milk for 1 h before incubation overnight with primary rabbit anti- human IL-1β cleaved (1:300) (Santa Cruz Technology) or rabbit-anti-human NLRP3 (1:500) (Cell Signaling Technology) or mouse-anti-human caspase-4 (1:500) Cell Signaling Technology), or for 1 h with mouse anti-human β-actin (Sigma Aldrich) (1:20,000) antibodies. After washing five times in TBS-T, membranes were revealed using peroxidase-conjugated secondary antibodies (Vector) (1:10,000) or streptavidin (R&D) (1:200).

### Quantification of inflammatory mediators in blood

The concentrations of the proteins platelet factor 4 (PF4/CXCL4), soluble P-selectin (sPselectin), macrophage migration inhibitory factor (MIF) and the eicosanoids thromboxane B_2_ (TXB_2_) and 12-hydroxyeicosatetraenoic acid (12-(S)-HETE) were measured in plasma from patients or healthy volunteers, or in supernatant from *in vitro* infected platelets, using standard commercially available enzyme-linked immunosorbent assay kits according to the manufacturer’s instructions (R&D Systems, Cayman Chemicals and ENZO).

### Virus, infections and virus titration

CHIKV (Asian strain) was kindly donated by Dr. Amilcar Tanuri and propagated in the Vero cells at an MOI of 0.1. Infection was carried out for 1 h at 37°C. The residual virus particles next were removed by washing with phosphate-buffered saline (PBS), and the cells were cultured for 2 to 5 days. After each period, the cells were lysed by freeze-thawing and centrifuged at 1,500 g at 4°C for 20 min to remove cellular debris. The Plaque-forming Assay was performed for virus titration in VERO E6 cells seeded in 24-well plates. Cell monolayers were infected with different dilutions of the supernatant containing the virus for 1h at 37˚C. The cells were overlaid with high glucose DMEM containing 2% FBS and 2.4% carboxymethylcellulose. After 3 days, the cells were fixed with 10% formaldehyde in PBS for 3h. The cell monolayers were stained with 0.04% crystal violet in 20% ethanol for 1h. The viral titer was calculated from the count of the plaques formed in the wells corresponding to each dilution and expressed as plaque forming unit per mL (PFU/mL). For *in vitro* analysis, 1.10^9^ platelets per milliliter from healthy donors diluted in medium 199 were infected with CHIKV for three hours (at 37°C in a 5% CO_2_ atmosphere), using the multiplicity of infection of 0.1 or 1 PFU/platelet. After 3 h of infection, the samples were centrifuged at 600 x g for 10 minutes and the supernatant was stored at -80°C for later quantification. Thrombin (1U/mL) was used as an experimental positive control, and Vero E6 cell medium maintained for the same culture time at the same dilution used to reach the desired MOI as a negative control. (Mock).

### Statistical analyses

All statistical analyses were performed using GraphPad Prism software (version 8.0.0, San Diego, CA). Comparisons between groups were performed using the student t test for parametric distributions, and the Mann-Whitney U test for nonparametric distributions. Results are presented as floating bars with median and quartiles. In all analyses, p<0.05 was considered statistically significant.

## Results

### Chikungunya fever triggers platelet activation

**Figure 1:**
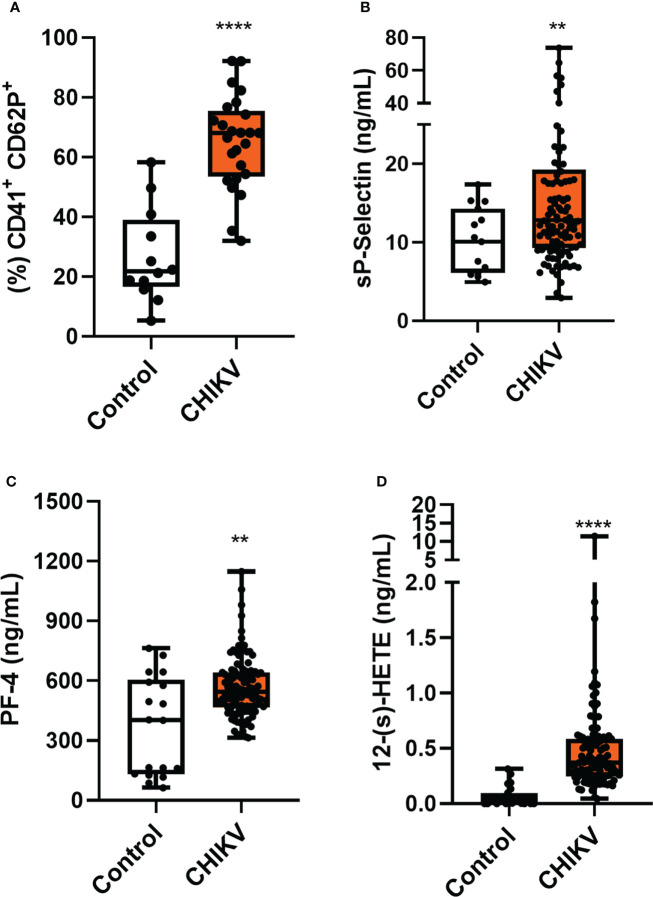
Chikungunya infection leads to platelet activation. **(A)** Analysis of CD62P expression on platelet surface by flow cytometry was performed in 25 patients and 12 controls, chosen randomly; **(B)** Plasma quantitation of the soluble molecule of CD62P; **(C)** Plasma quantitation of platelet factor 4 (PF-4); and **(D)** Plasma quantitation of 12-(S)-HETE. **(B–D)** Samples were collected from 132 (CHIKV) and 25 healthy volunteers (Control). The symbol ** means p<0.01 and the **** means p<0.0001.

The clinical profile of chikungunya patients (CHIKV) is described in **Table 1**. The importance of platelet activation has already shown in other viral infections, especially dengue (30–32).We started our investigation analyzing whether platelets would be activated by chikungunya virus (CHIKV) infection when compared to healthy donors (Control). We performed the analysis of P-selectin (CD62P) in isolated platelets. P-selectin is an important adhesion molecule that is stored in alpha granules and participates in platelet interactions with endothelial cells, monocytes, neutrophils and lymphocytes (21). As shown in **Figure 1A**, Chikungunya fever increased P-selectin (CD62P) expression in platelets and the amount of soluble P Selectin (sP-selectin) in plasma from patients compared to healthy donors (**Figure 1B**). This data indicates that CHIKV infection leads to platelet activation in patients.

**Table 1:** Characteristics of Chikungunya patients and control donors.

Characteristics^1^	Control (25)	Patients (132)
Age, years	36 (28.5-45)	44 (33-56)
Sex, male	11 (44%)	61 (46%)
Systemic arterial hypertension	–	32(24%)
Onset of symptoms (days)	–	2 (2-3)
Viral load (copies/mL)	–	69.95 (0.08-21.387)
Clinical Symptoms (acute phase)		
Fever	–	124 (93%)
Rash	–	43 (32%)
Headache	–	94 (71%)
Retro-orbital pain	–	52 (39%)
Myalgia	–	93 (70%)
Arthralgia	–	120 (90%)
Prostration	–	60 (45%)
Conjunctivitis	–	2 (1.5%)
Limb edema	–	27 (20%)
Nausea and vomiting	–	40 (30%)
Pain on Palpation	–	7 (5.3%)
Arthritis	–	2 (1.5%)
Weakness in Hands and Legs	–	34 (25.75%)
Numbness	–	14 (10.6%)
Swallowing Difficulty	–	2 (1.5%)

^1^Numerical variables are represented as the median and the interquartile range, and qualitative variables are represented as the number and the percentage.

To confirm increased platelet activation in CHIKV infection, we analyzed the release of inflammatory mediators produced mainly/exclusively by platelets in patients’ plasma. Like the CD62P molecule, platelet factor 4 (CXCL-4/PF-4) is stored in platelet alpha granules and is rapidly released once platelets are activated. PF-4 is a chemokine secreted exclusively by platelets and megakaryocytes. We observed in **Figure 1C** that PF4 release was increased in plasma from Chikungunya patients when compared to the control. When platelets are activated, arachidonic acid (AA) is released from membrane phospholipids by phospholipase A_2_ (PLA_2_). The AA in platelets is metabolizes by two alternative metabolic pathways, the cyclooxygenase (COX) and the 12-lipoxygenase (12-LOX). The 12-LOX activity metabolizes AA to 12-hydroperoxy-5,8,10,14-eicosatetraenoic acid S enantiomer (12-(s)HpETE), which is rapidly reduced to 12-hydroxyeicosatetraenoic acid (12-(s)HETE) (33). As observed in **Figure 1D**, patients’ plasma had a higher concentration of 12-HETE when compared to control. Collectively, these results support the hypothesis that platelets are activated during chikungunya virus infection.

### Chikungunya fever triggers inflammasome activation in platelets

**Figure 2:**
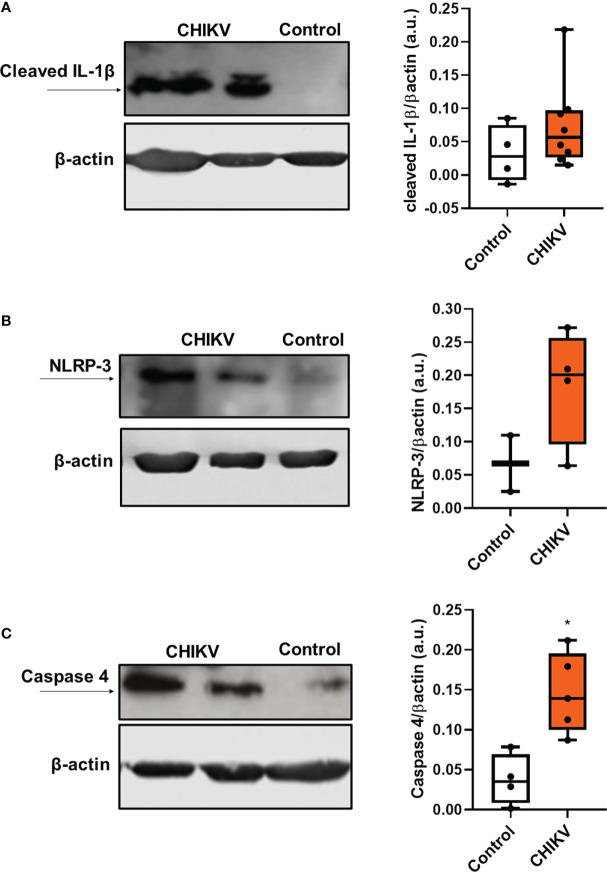
Infection with chikungunya virus leads to inflammasome activation in platelets. **(A)** Western blot analysis of cleaved IL-1 β and β-actin expression in platelets isolated from four control subjects and eight CHIKV patients and graph demonstrating band densitometry comparing to CHIKV with Control (NS, p=0.2828); **(B)** Western blot analysis of NLRP-3 and β-actin expression in platelets isolated from three control subjects and four CHIKV patients and graph demonstrating band densitometry comparing to CHIKV with Control (NS, p=0.2667); **(C)** Western blot analysis of caspase 4 and β-actin expression in platelets isolated from four control subjects and five patients with CHIKV; and graph demonstrating band densitometry with *P ≤ 0.05 comparing to CHIKV with Control.

Recent studies have shown that platelets, which are anucleated cells, possess pre-messenger RNA and mRNA, being capable of mRNA splicing, translation and protein synthesis, including IL-1β (34). Platelets constitutively express the components of the NLRP3 and ASC inflammasome and can use them to assemble a functional inflammasome, activating caspase 1 to process IL-1β (31). Using purified platelets from patients with CHIKV and Controls, we analyzed the expression of cleaved IL-1β, the active form of the protein. As noted in **Figure 2A**, we observed a prominent 17-kD size band corresponding to cleaved IL-1β in platelets from Chikungunya patients compared to control. In addition, we also observed an increase in the expression of NLRP3 (**Figure 2B**). This data suggests that the infection leads to modulation of IL-1β and NLRP3 protein expression, and that platelets present the active machinery for IL-1β processing. Inflammasomes are multiprotein complexes of the innate immune system that orchestrate development of inflammation by activating the secretion of the proinflammatory cytokines IL-1β and IL-18. Recently, it was demonstrated that caspase 4 is part of a non-canonical inflammasome pathway that activates NLRP3 and generates IL-1β processing. In our work, we observed that caspase 4 expression was also increased in platelets during CHIKV infection (**Figure 2C**).

### Platelet infection with CHIKV *in vitro* partially reproduces the phenotype in patients

**Figure 3:**
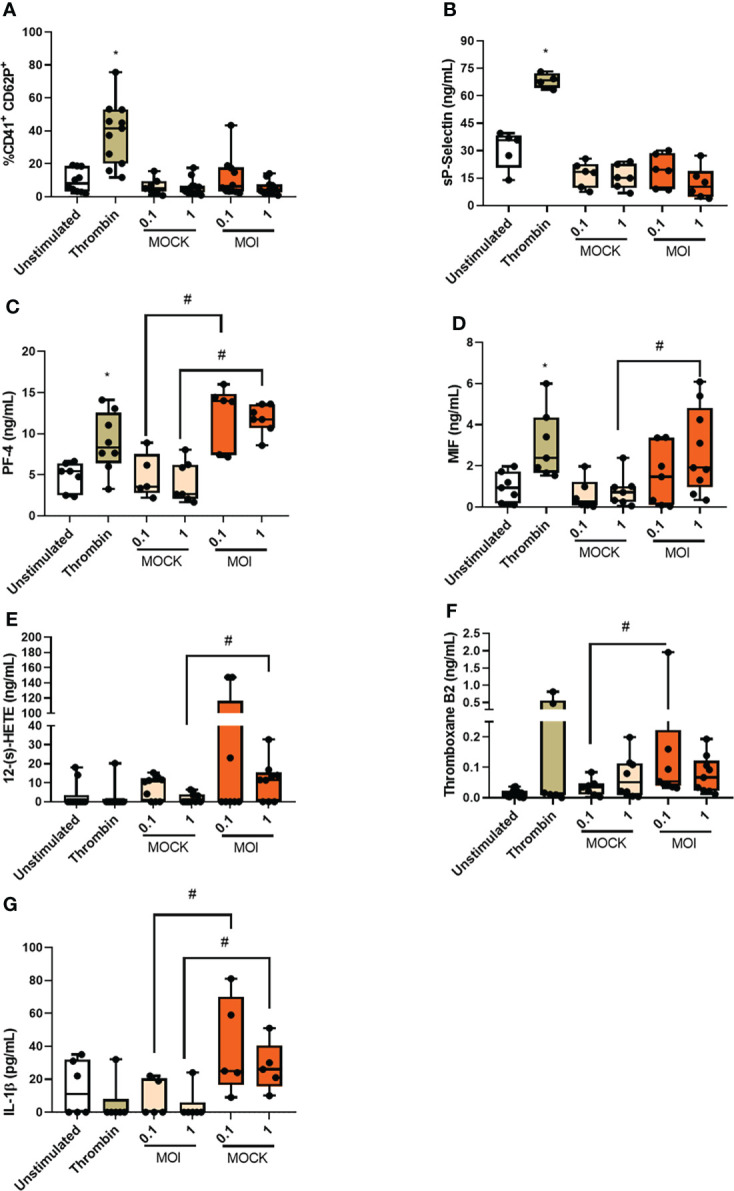
Chikungunya virus infection leads to platelet activation *in vitro.* Platelets from healthy donors were infected with chikungunya virus at MOIs of 0.1 and 1 for three hours. **(A)** Analysis of CD62P expression on platelet surface by flow cytometry; **(B)** Quantification of soluble CD62P in the supernatant of infected platelets*;* **(C)** Quantification of platelet factor 4 (PF-4) in the supernatant of infected platelets; **(D)** Quantification of Macrophage migration inhibitory factor (MIF) in the supernatant of infected platelets; **(E)** Quantification of 12-(s)-HETE in the supernatant of infected platelets; **(F)** Quantification of Thromboxane in the supernatant of infected platelets and **(G)** Quantification of IL-1β in the supernatant of infected platelets. *P ≤ 0.05 comparing to CHIKV with Control and ^#^P ≤ 0.05 comparing to CHIKV with MOCK.

To better understand platelet activation during CHIKV infection, we performed *in vitro* analyses. Therefore, isolated platelets from healthy donors were infected with CHIKV (MOI = 0.1 or 1) for three hours and platelet activation was assessed. We do not observe increases in the expression of CD62P on the platelet surface (**Figure 3A**) or its soluble form in the supernatant (**Figure 3B**), when compared to the uninfected controls (uninfected platelet and Mock). In this group of experiments, we used thrombin as positive control that leads to increased expression and release of CD62P (**Figures 3A, B**). We then evaluated whether CHIKV infection could lead to the release of cytokines present in platelet alpha granules. We observed that the two MOIs analyzed led to an increase in PF-4 release, as did the positive control (**Figure 3C**). We also analyzed the release of macrophage migration inhibitory factor (MIF), which is a relevant cytokine in platelet activation by the DENV (35), and observed that CHIKV infection at the MOI of 1 and the positive control induced an increase in MIF secretion (**Figure 3D**). Our next step was to analyze the release of platelet-derived lipid mediators. We observed increased release of 12-(s)-HETE by platelets infected at MOI of 1 (**Figure 3E**) and thromboxane release at the MOI of 0.01 (**Figure 3F**). The positive control did not lead to consistent release of either mediator. To understand if the processing and release of IL-1β would occur in platelets infected with CHIKV *in vitro*, we measured the release of IL-1β in the supernatant. We observed that the two MOIs tested led to the release of IL-1β while the positive control did not (**Figure 3G**). These data show that platelet infection with CHIKV *in vitro* partially reproduces the activation phenotype observed in patients. Based on the differences observed between *in vivo* and *in vitro* activated platelets, we suggest that some of the phenomena observed in the patients that are not induced by the *in vitro* infection model may be generated through indirect platelet activation by the inflammatory milieu and/or by direct activation and reprogramming of megakaryocytes.

### Increased platelet activation converges with chronic cases of chikungunya fever

**Figure 4:**
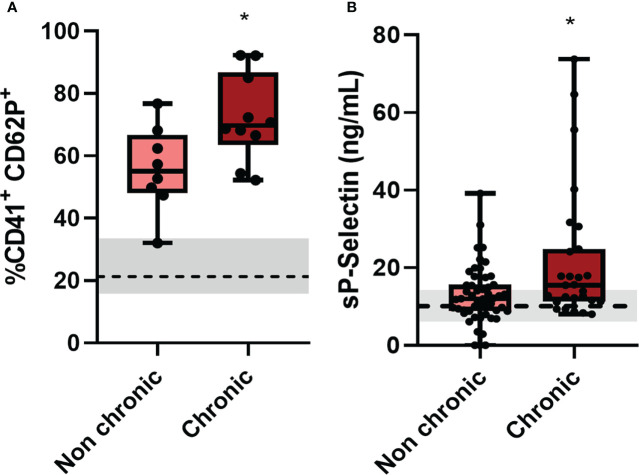
Patients who progressed to chronic conditions of CHIKV had greater platelet activation. **(A)** Patients who progressed to a chronic condition had higher expression of CD62P on the surface of platelets by flow cytometry, of the 25 patients analyzed, 8 did not progress to a conical condition and 10 became chronic, the other 7 did not have contact; and **(B)** Plasma quantitation of the soluble molecule of CD62P of patients who progressed to a chronic condition, of the 80 patients contacted, 51 did not progress to a chronical condition and 27 became chronic, the other 2 did not obtain quantification. *P ≤ 0.05 comparing non-chronic to chronic condition.

The most noticeable clinical feature of chikungunya fever is that after the acute phase a high proportion of infected individuals experience chronic incapacitating arthralgia, which can last for months to years (36). To assess whether platelet activation in the acute phase of the disease could be related to the chronicity of the cases, we contacted patients eighteen months after clinical recovery and asked if they still felt of joint pain/arthralgia and/or muscle pain (**Table 2**). With this information, we divided Chikungunya patients in two groups: Those who no longer had any joint pain/arthralgia and/or muscle pain (non-chronic); and those who remained with joint pain/arthralgia and/or muscle pain (chronic). Among the 132 patients analyzed in this study, 51 progressed to a chronic condition and 29 had no symptoms related to chikungunya infection after the acute phase. We observed that those who progressed to a chronic condition of the disease had higher platelet activation (**Figure 4A**) and increased release of soluble p-selectin (**Figure 4B**) than those who did not progress to chronic disease. In the graphs, we demarcate the interquartiles with a gray rectangle and the dotted line corresponds to the median of the values referring to the controls. These data suggest that exacerbated platelet activation in the acute CHIKV infection could contribute to and may predict the chronicity of patients.

**Table 2:** Characterization of the population regarding the symptoms presented after 1 year of diagnosis.

Characteristics^1^	Non chronic (51)	Chronic (29)
Age, years	38 (30.5-55)	48 (42-57)
Sex, male	31 (60%)	8 (27.6%)
Joint and/or muscle pain	0 (0%)	29 (100%)

^1^Numerical variables are represented as the median and the interquartile range, and qualitative variables are represented as the number and the percentage.

## Discussion

Platelets are well known cells for their hemostatic activities that are currently recognized by their important functions in the immune response (21), participating in the pathophysiology of several diseases including arthritis (37) and infectious diseases as dengue (30, 32–36, 38), COVID-19 (39–41) and malaria (42–45). Ultrastructural studies have demonstrated that chikungunya virus is associated with human platelets by becoming entrapped in platelet aggregates, and some of the platelets showed features of degranulation and lysis during this process (46, 47). Platelets from CHIKV patients and *in vitro* infected platelets in the present study also showed features of platelets activation, even though some features were exclusive of platelets from patients.

We have observed increased platelet P-selectin surface expression and increased soluble P-selectin released in the plasma from Chikungunya patients. P-selectin is a glycoprotein stored in platelet α-granules that is translocated to the surface and released in suspension during platelet activation. P-selectin surface expression is increased in patients with dengue, and is higher in patients presenting warning signs and severe dengue syndromes compared to mild dengue (26). P-selectin is the main adhesion molecule responsible for platelet interaction with monocytes (21, 48) and circulating platelet-monocyte aggregates are increased in dengue-infected patients (32). P-selectin and PF-4 are also important markers of platelet activation in other viral infections such as SARS-CoV-2 and HIV. In COVID-19 it has been shown that P-selectin is essential for platelet-monocyte binding and consequent tissue factor expression, leading to a amplification of thromboinflammatory response (39, 40). Also in COVID-19, several studies have shown a correlation between increase PF-4 levels and severe cases, as well as in cases of post-vaccine thrombosis (40, 49). Regarding HIV infection, it is observed that platelet activation with increased P-selectin expression is persistent even with antiretroviral therapy, being a complicating factor because it participates in thrombus formation and increases the risk of developing cardiovascular diseases (28, 50). 12-HETE is a product of arachidonic acid metabolism that has been reported as a major modulator of vascular and joint inflammation during the pathophysiology of sterile and infectious diseases. In our study we observed increased 12-(S)-HETE levels in the plasma of CHIKV patients. Platelet activation and 12-HETE synthesis were previously related to activation and infiltration of neutrophils to the inflamed joints of rheumatoid arthritis patients (51). Since rheumatoid arthritis is also a complication of the chronic phase of CHIKV infection, these data are suggestive of the participation of platelet activation and 12-HETE release in Chikungunya fever arthropathy. Accordingly, platelet activation at the acute phase was higher in patients that progressed with joint and muscle pain in the chronic phase in the present study. In addition, increased 12-(S)-HETE synthesis was related to patients with hypertension (52) and increased pulmonary permeability in models of sterile inflammation (53), however we could not detect differences in plasma 12-HETE between patients with non-chronic/chronic joint or muscle pain

Platelets have already been shown to have the machinery to perform post-transcriptional splicing and IL-1β synthesis (34). We have previously shown that during dengue there is a significant positive correlation between thrombocytopenia and IL-1β release (54) and that dengue infection leads to assembly of NLRP3 inflammasomes, activation of caspase 1, and caspase 1–dependent IL-1β secretion in dengue (31). In the present work, we confirm the accumulation of cleaved IL-1β and NLRP3 proteins in platelets from patients with CHIKV infection. Inhibiting IL-1β signaling in CHIKV-infected mice conferred protection from bone loss and consequent improvement in arthritogenic conditions (55). NLRP3-dependent inflammasome assembly and consequent IL-1β activation has been shown to be pathogenic in several viral infections contributing to pulmonary fibrosis in cytomegalovirus infected mice (56) and in patients with acute-on-chronic hepatitis B liver failure (57). In infections with influenza A virus, however, activation of NLRP3-dependent inflammasome complex contributes to protective responses and to ameliorate illness (58). Despite the contradictory response depending on the type of viral infection, the importance of NLRP3-dependent inflammasome in various viral infections is contiguous and therefore an interesting target for manipulation aiming at improved disease outcomes, including in CHIKV infection. There are no previous reports on the role of caspase 4 in platelets, in our work we demonstrated increased caspase 4 expression in patients with CHIKV. The increase in caspase together with NALP3 and IL-1β cleavage suggests that non-canonical inflammasome assembly occurs in these platelets. Caspase 4 is a caspase not related to apoptosis and, like caspase 1, it is considered an inflammatory caspase. Activation of noncanonical inflammasome can activate the NLRP3-caspase-4-mediated processing and secretion of IL-1β and IL-18, and induces the inflammatory cell death, pyroptosis, *via* gasdermin D cleavage and activation (59–62). Increased caspase 4 activation in A549 epithelial cells has been linked to growth restriction of *B. pseudomallei* (63). However, we cannot rule out the role of caspase 1 in IL-1β processing in CHIKV platelets, and therefore more experiments are needed to understand the role of each protein in platelet inflammasome engagement during CHIKV infection.

Although our experiments indicate strong platelet activation during chikungunya fever, our *in vitro* infection model reproduced only part of the patients’ platelet phenotype. We observed that CHIKV induces the release of inflammatory mediators, including cytokines and eicosanoids, important in various immunoregulatory processes and diseases, but not in the translocation of P-selectin to the cell surface or its release as a soluble molecule. We can speculate to explain this difference that CHIKV infection can peripherally generate the release of several classic stimuli, such as ADP, fibrinogen, thrombin (which we used as a positive control), as well as inflammatory mediators such as interleukin 6 (IL-6) or Tumor necrosis factor (TNF) and coagulation factors aiding greater platelet activation (64). The pathways that platelets respond to the pathogen can also vary *in vitro* and *in vivo* according to the receptor used. For example, in presence of antibodies platelets would interact with CHIKV through the FcγRIIA receptor (CD32a), a low affinity Fc receptor for the immunoglobulin G constant region that recognizes immune complexes with IgGs. Recognition through this receptor triggers intracellular signaling events that lead to platelet activation and aggregation, which is a very common event in bacterial infections but has also been reported in infections generated by dengue and H1N1 influenza viruses (64–69). In our *in vitro* experiments, when using purified platelets diluted in culture medium, these other elements of the infection microenvironment that may be participating in platelet response are missing. However, we cannot rule out that during infection, CHIKV is also directly stimulating megakaryocytes, as has been seen in other infections, such as dengue virus, SARS-CoV-2 and HIV, with alterations in the maturation and development of the megakaryocyte, in the production of platelets, permissiveness to viral replication, formation of infectious viral particles and thromboinflammation, potentially influencing the observations we have in patients (70–77). Further studies are still needed to fully understand the mechanisms of platelet and megakaryocyte activation and their contributions to the pathophysiology of chikungunya fever.

A high proportion of individuals infected with CHIKV experience intense and limiting joint pain (arthralgia), which fails to return to healthy conditions after the acute phase, and which clinical cure can take weeks to months, thus affecting the quality of life. Progression to the chronic phase appears to be independent of the viral strain and location of the outbreak. Some studies have positively correlated factors such as increasing age, time of acute disease persistence, female gender, pre-existing rheumatologic conditions, high viral titers and high level of anti-CHIKV antibodies with the persistence of the disease to a chronic phase (78, 79). High levels of interleukin 12 have been linked to both the acute and chronic phases of the disease (16). Analyzes performed in chronic patients identified a large infiltrate of leukocytes, including TCD8, TCD4, NK cells and macrophages in biopsies of muscle tissue, joint-associated tissue and synovial fluid during chronic CHIKV disease (16, 80). Increased cytokines and chemokines such as IL-6, GM-CSF, IL-1a, IL-15, CXCL9 and CXCL10 and complement system components such as C3 were also observed from 6 to 36 months after infection in patients with chronic disease compared to with retrieved controls (12, 81, 82). Increased platelet activation in patients that have evolved with chronic arthralgia and myalgia observed in this work is suggestive of an active participation of platelets in the generation of immune mechanisms related to chronic progression. Our observation that patients who progressed to a chronic condition presented higher platelet activation in the acute phase, suggests platelets as another immunological component predisposing to chronicity. New studies are still needed to investigate whether platelets remain activated or predisposed to be activated in these patients who have progressed to a chronic condition compared to the healed patients.

In summary, we demonstrated for the first time that platelets are activated in Chikungunya virus infection, and that this activation can lead to NLRP3 inflammasome formation and IL-1β processing besides the release of inflammatory eicosanoids, cytokines and chemokines. Therefore, platelet activation contributes to inflammatory amplification during acute phase, which may be implicated in the progression to chronic disease in Chikungunya. All these activation-dependent responses and clinical associations highlight a role for platelets in pathophysiological mechanisms of this arboviral disease that continues as a major public health problem.

## Data availability statement

The raw data supporting the conclusions of this article will be made available by the authors, without undue reservation.

## Ethics statement

The studies involving human participants were reviewed and approved by The Institutional Review Board (IOC/FIOCRUZ 42999214.1.1001.5248). The patients/participants provided their written informed consent to participate in this study.

## Author contributions

IA-Q, FB, EH and PB conceived the study. IA-Q, MC, AM, AC, SD, DO, VS, and JS performed experiments and analyzed data. IA-Q wrote the manuscript. IA-Q, FB, EH and PB performed experimental design, manuscript review and supervised the study. IT and AN participate in patient inclusion and clinical data analysis. TMLS performed experimental design, molecular diagnostic and supervised the study. All authors reviewed the manuscript. All authors contributed to the article and approved the submitted version.

## Funding

This work was supported by Conselho de Desenvolvimento Científico e Tecnológico (CNPq), Fundação de Amparo à Pesquisa do Estado do Rio de Janeiro (FAPERJ) and Programa de Núcleos de Excelência (Pronex). Coordenação de Aperfeiçoamento de Pessoal de Nível Superior (CAPES) Funding was also provided by CNPq and FAPERJ through the National Institutes of Science and Technology Program (INCT) on Diseases of Neglected Populations.

## Conflict of interest

The authors declare that the research was conducted in the absence of any commercial or financial relationships that could be construed as a potential conflict of interest.

## Publisher’s note

All claims expressed in this article are solely those of the authors and do not necessarily represent those of their affiliated organizations, or those of the publisher, the editors and the reviewers. Any product that may be evaluated in this article, or claim that may be made by its manufacturer, is not guaranteed or endorsed by the publisher.
